# miR-1260b Activates Wnt Signaling by Targeting Secreted Frizzled-Related Protein 1 to Regulate Taxane Resistance in Lung Adenocarcinoma

**DOI:** 10.3389/fonc.2020.557327

**Published:** 2020-11-05

**Authors:** Jin Ren, Deqiang Wang, Hanpeng Huang, Xiaoqin Li, Xiufen Zhuang, Jian Li

**Affiliations:** ^1^Department of Medical Oncology, Affiliated Hospital of Jiangsu University, Zhenjiang, China; ^2^Department of Pulmonary Medicine, Affiliated Hospital of Jiangsu University, Zhenjiang, China

**Keywords:** lung adenocarcinoma, taxane, chemoresistance, Wnt signaling, SFRP1, miR-1260

## Abstract

**Objectives:** MicroRNAs (miRNAs) have been demonstrated to contribute to carcinogenesis; however, their association with tumor chemoresistance is not fully understood. In this study we aimed to investigate the molecular mechanisms involved in resistance to taxane-based chemotherapy in lung adenocarcinoma (LAD).

**Methods:** We established paclitaxel-resistant A549 cells (A549/PTX) and docetaxel-resistant H1299 cells (H1299/DTX). In order to hit the mark, we employed multiple methods including qRT-PCR, western blotting analysis, loss/gain-of-function analysis, luciferase assays, drug sensitivity assays, animal experiment, wound-healing assay, and invasion assay.

**Results:** Bioinformatics analysis and a luciferase reporter assay revealed that secreted frizzled-related protein 1 (SFRP1) is a direct target of miR-1260b. By qRT-PCR analysis, we found that miR-1260b was significantly upregulated in taxane-resistant cells as compared to parental cells. Suppression of miR-1260b reversed the chemoresistance of human LAD cells to taxanes both *in vitro* and *in vivo*, whereas ectopic miR-1260b expression decreased the sensitivity of parental LAD cell lines to taxanes. Downregulation of miR-1260b expression inactivated the Wnt signaling pathway and reversed the epithelial-mesenchymal transition (EMT) phenotype of taxane-resistant LAD cells. In clinical tumor tissue samples, high miR-1260b expression was detected in tumors of non-responding patients treated with taxane-based chemotherapy and was associated with low SFRP1 expression and poor prognosis.

**Conclusions:** Our findings reveal that targeting of the miR-1260b/SFRP1/Wnt signaling axis might provide a novel strategy for overcoming chemotherapy resistance in LAD.

## Introduction

Lung cancer is a leading cause of cancer-related deaths due to its high prevalence, aggressiveness, and poor prognosis (1). Lung adenocarcinoma (LAD) is the most common histological type of lung cancer and is usually diagnosed at an advanced stage (2). Despite the development of cancer treatments and the introduction of new technologies, chemotherapy remains one of the main treatment methods to improve survival and prognosis in LAD patients (3). Taxanes, such as paclitaxel and docetaxel, are common chemotherapy agents used to treat many types of cancers, including advanced LAD and other solid tumors (4). However, chemoresistance is a major obstacle in taxane therapy. The identification of the molecular mechanisms involved in LAD chemoresistance is a critical step toward the development of novel therapeutics.

Substantial evidence indicates that the Wnt/β-catenin pathway plays a vital role in tumor cell survival, metastasis, and chemoresistance (5). Secreted frizzled-related protein 1 (SFRP1), a 35-kDa secreted glycoprotein, is an extracellular signaling molecule used to antagonize Wnt signaling (6). We previously found that SFRP1 is significantly downregulated in paclitaxel-resistant A549 cells (A549/PTX) when compared with parental A549 cells. SFRP1 might act as a tumor suppressor to reverse taxane resistance of LAD cells by inactivating Wnt signaling, as indicated by both *in vitro* and *in vivo* experiments (7). Nevertheless, the mechanisms that regulate the loss of SFRP1 remain to be investigated.

MicroRNAs (miRNAs) regulate ~30% of human gene expression (8). miRNAs can control gene expression by directly binding to the 3′-untranslated region (3′-UTR) of target mRNAs, which leads to degradation of the mRNA transcript or inhibition of the protein translation process (9). miRNAs play important roles in various biological and pathological processes, such as cell differentiation, proliferation, and carcinogenesis (10). Some recent studies have highlighted that miRNAs can induce chemoresistance in various tumors by altering gene expression (11, 12). On the basis of this idea, we hypothesized that miRNAs might be involved in the loss of SFRP1 and taxane resistance of LAD cells by affecting Wnt pathway activity.

In the current study, we report for the first time that SFRP1 is a direct target of miR-1260b in LAD cells. Specifically, we identify miR-1260b as a strongly upregulated miRNA in paclitaxel-resistant LAD cells. MiR-1260b-dependent downregulation of SFRP1, which contributes to the activation of Wnt/β-catenin signaling, modulates the sensitivity of LAD cells to multiple antitumor drugs both *in vivo* and *in vitro*. These findings confirm the importance of miR-1260b in the development of chemoresistance and provide the first evidence for miR-1260b-dependent regulation of chemoresistance.

## Materials and Methods

### Cell Culture and Treatment

The human lung adenocarcinoma cell lines H1299 and A549 were obtained from the Shanghai Institute of Cell Biology (Shanghai, China). Paclitaxel-resistant A549 cells (A549/PTX) were a gift from Dr. Liu (Nanjing Gulou Hospital, Jiangsu, China) and maintained in the presence of 200 μg/L paclitaxel. Docetaxel-resistant H1299 cells (H1299/DTX) were established by sequential exposure to docetaxel at increasing concentrations as described previously, and were maintained in the presence of 50 μg/L docetaxel (13). All cells were maintained in RPMI-1640 medium (Gibco, Grand Island, NY, USA) supplemented with 10% fetal bovine serum (FBS, Gibco, USA), penicillin, and streptomycin (Invitrogen, Shanghai, China) at 37°C in an atmosphere of 5% CO_2_.

### Plasmid Construction and 3′-UTR Luciferase Assa*y*

Putative miR-1260b-binding sites in the SFRP1 gene were identified using public prediction software (http://targetscan.org). The potential sequences were amplified by PCR from A549 cell genomic DNA and cloned downstream of the firefly luciferase reporter gene in the pLUC vector (Promega, Madison, WI, USA). The primer sequences used for plasmid construction are listed in Supplementary Table 1. Mutant reporters were generated by GeneChem (Shanghai, China). A549 Cells were cotransfected with pLUC-SFRP1-3′-UTR or pLUC-SFRP1-3′-UTR mutant plasmid, miR-NC or miR-1260b precursor, and renilla. After 48 h of transfection, luciferase activity was assessed using the Dual-Glo Luciferase Assay System (Promega, USA).

### Cell Transfection

The miR-1260b precursor (PmiR-1260b), miR-1260b inhibitor (AmiR-1260b), and their corresponding negative controls (NC), green fluorescent protein (GFP)-empty (Lv-NC) lentiviral vector, and GFP-miR-1260b-knockdown (Lv-anti-miR-1260b) lentiviral construct were all obtained from GeneChem (Shanghai, China). Lentiviral infection was performed based on the protocols. Briefly, cells were planted into 12-well plates (1 × 10^5^ cells/well), and then infected with lentivirus at a multiplicity of 10 plaque-forming units/cell. After 72 h of infection, cells were screened with puromycin. Survived cells were selected and prepared for subsequent experiments.

### Quantitative Real-Time (qRT)-PCR

Total RNA was isolated from tissues and cells using TRIzol reagent (Invitrogen, USA) and reversely transcribed into cDNA using a PrimeScript RT Reagent Kit (Takara, Otsu, Japan) following the vendor's recommendations. All PCR reactions were run in triplicate in a PRISM 7900 Sequence Detection System (Thermo Scientific, Waltham, MA, USA) using the miScript SYBR Green PCR kit (Takara). Sequence information for all primers is shown in Supplementary Table 1. The expression of miR-1260b or genes was calculated relative to that of U6 or GAPDH as an endogenous control. Fold changes in expression were calculated using the 2^−ΔΔCt^ method.

### Western Blotting

Equal amounts of cell lysates were resolved on a 10% sodium dodecyl sulfate polyacrylamide gel and blotted onto a polyvinylidene fluoride membrane (Millipore, Billerica, MA). The membranes were blocked with 5% BSA for 1 h and probed with primary antibodies to SFRP1 (1:250, Abcam, Cambridge, MA, USA), β-catenin (1:1000, bioWORLD, Dublin, OH, USA), p-GSK3β (1:1000, bioWORLD), GSK3β (1:1000, bioWORLD), cyclin D1 (1:1000, Cell Signaling Technology, Danvers, MA, USA), or c-Myc (1:1000, Santa Cruz Biotechnology, Santa Cruz, CA, USA). After incubation with horseradish peroxidase-conjugated goat anti-rabbit or goat anti-mouse antibody (1:2000, Abcam Inc., Cambridge, UK), protein bands were visualized using ECL substrate (Thermo Scientific, USA).

### Cell Viability Assay

Cells were seeded into 96-well plates (3 × 10^3^ cells/well) and incubated with various concentrations of drugs for 72 h. Cell viability was assessed by 3-(4,5-dimethylthiazol-2-yl)-2,5-diphe-nyltetrazolium bromide (MTT, Sigma-Aldrich, St. Louis, MO) assays, as previously described (7).

### Flow-Cytometric Cell Cycle Analysis

Cells were collected and fixed in 70% ethanol at −20°C. After incubation with propidium iodide (PI)/RNase staining solution (MultiSciences, Hangzhou, China), cell cycle analysis was conducted by flow cytometry in a FACScan instrument (BD Biosciences, San Jose, CA, USA), as previously described (7).

### Apoptosis Analysis

The apoptotic rate was measured using an Annexin V-FITC/PI Apoptosis Detection Kit (KeyGen Biotech, Nanjing, China) according to the manufacturer's instructions. Cells were suspended in 0.5 ml binding buffer and stained with 5 μl annexin V and 5 μl PI. The analysis was performed in a BD FACSCanto II (BD Biosciences, USA) flow cytometry and FlowJo software.

### Colony Formation Assay

Cells were seeded into 6-well plates (1,000 cells/well) and cultured in RPMI 1640 medium for 14 days. Then, the cells were fixed with methanol and stained with 1% crystal violet solution to visualize colonies for counting.

### TCF/LEF Reporter Assay

Wnt/β-catenin signaling pathway activity was evaluated by TCF/LEF reporter assays. TOPflash, FOPflash, and pRL-SV40 were all purchased from Upstate (Lake Placid, NY, USA). Cells were cotransfected with miRNA-1260b precursor (or inhibitor), TOPflash (or FOPflash), pcDNA3.1/SFRP1 (or pcDNA3.1), and pRL-SV40. Cell lysates were analyzed for firefly and renilla luciferase activities using the Dual-Luciferase Reporter Assay System (Promega, USA) according to the manufacturer's instructions. Each assay was conducted in triplicate wells, and data are reported as the mean of three independent experiments.

### Wound Healing Assay

5 × 10^5^ cells were grown to confluent monolayer in 6-well plates. After serum starvation in serum-free medium for 24 h, the monolayer cells were scratched using 200 μl tips to create a denuded zone (gap) of constant width. The imagination of wounded areas was viewed under a microscope and photographed at the indicated time points (0, 24, and 48 h). The distances between the two edges of the scratched cells were measured and healing rate was calculated, as previously described (14).

### Invasion Assay

5 × 10^4^ cells in serum-free media were placed into the upper chambers of 8-μm pore size Transwell plates (Corning, MA, USA). The lower chambers were filled with RPMI-1640 media containing 10% FBS. After 48 h incubation, the migrated cells were fixed with methanol, stained with crystal violet (Beyotime Biotech., Jiangsu, China), and counted under a microscope. Each sample was measured in triplicate, and the experiment was performed at least three times.

### Animal Experiments

28 BALB/c athymic nude mice (male, 5–6-week-old, specific pathogen-free) were obtained from the animal center of Jiangsu University. All animal experiments were performed in accordance with protocols approved by the Institutional Animal Care and Use Committee of Jiangsu University. A549/PTX cells stably downregulating miR-1260b, H1299/DTX cells stably downregulating miR-1260b and negative control cells (NC) were inoculated subcutaneously into the right flanks with 7 mice per group, respectively. Tumor volume was measured using a caliper every other day, and calculated using the following equation: volume (mm^3^) = (length × width^2^)/2. When the mean tumor volume reached ~100 mm^3^, paclitaxel or docetaxel was intraperitoneally injected into A549/PTX or H1299/DTX xenograft mice with a concentration of 15 or 1 mg/kg every 3 days for three times in total, respectively. After 5 weeks, the mice were euthanized and tumor tissues were removed for H&E staining and proliferating cell nuclear antigen (PCNA) protein immunostaining analysis as previously described (15).

Stable miR-1260b knockdown A549/PTX cells were injected into the lateral tail vein. After 8 weeks, lung tissues were excised and analyzed by H&E staining. Blood was collected and red blood cells were removed. RNA from the remaining cells was extracted for real-time PCR. The relative concentration of circulating tumor cells was assessed based on human-specific GAPDH (hGAPDH) expression relative to mouse-specific GAPDH (mGAPDH) and human-miR-1260b (hmiR-1260b) expression relative to mouse- miR-1260b (mmiR-1260b) (14).

### Clinical Samples

A total of 38 patients with advanced LAD receiving chemotherapy at the Affiliated Hospital of Jiangsu University from January 2012 and December 2017 were enrolled in the study. Based on the Tumor-Node-Metastasis staging system, these cases were staged IIIB/IV and histologically diagnosed as having LAD with at least one measurable lesion. All patients received first-line chemotherapy comprising paclitaxel and cisplatin or paclitaxel and carboplatin every 3 weeks for a maximum of six cycles. The tumor response was assessed by medical image analysis and categorized based on Response Evaluation Criteria in Solid Tumors as complete response (CR), partial response (PR), stable disease (SD), or progressive disease (PD). This study was approved by the Ethics Committee of Affiliated Hospital of Jiangsu University.

### Statistical Analysis

Data are representative of at least three experiments. Significance was assessed by a two-tailed Student's *t*-test. The Kaplan-Meier method was used to estimate the probability of survival. All statistical analyses were performed using SPSS (version 16.0) and GraphPad (Version 5.0). A *p*-value < 0.05 was considered significant (^*^p < 0.05, ^**^p < 0.01).

## Results

### Parental A549 Cells and Paclitaxel-Resistant A549/PTX Cells Differ in Physiology and miR-1260b Directly Targets SFRP1 in LAD Cells

**Figure 1:**
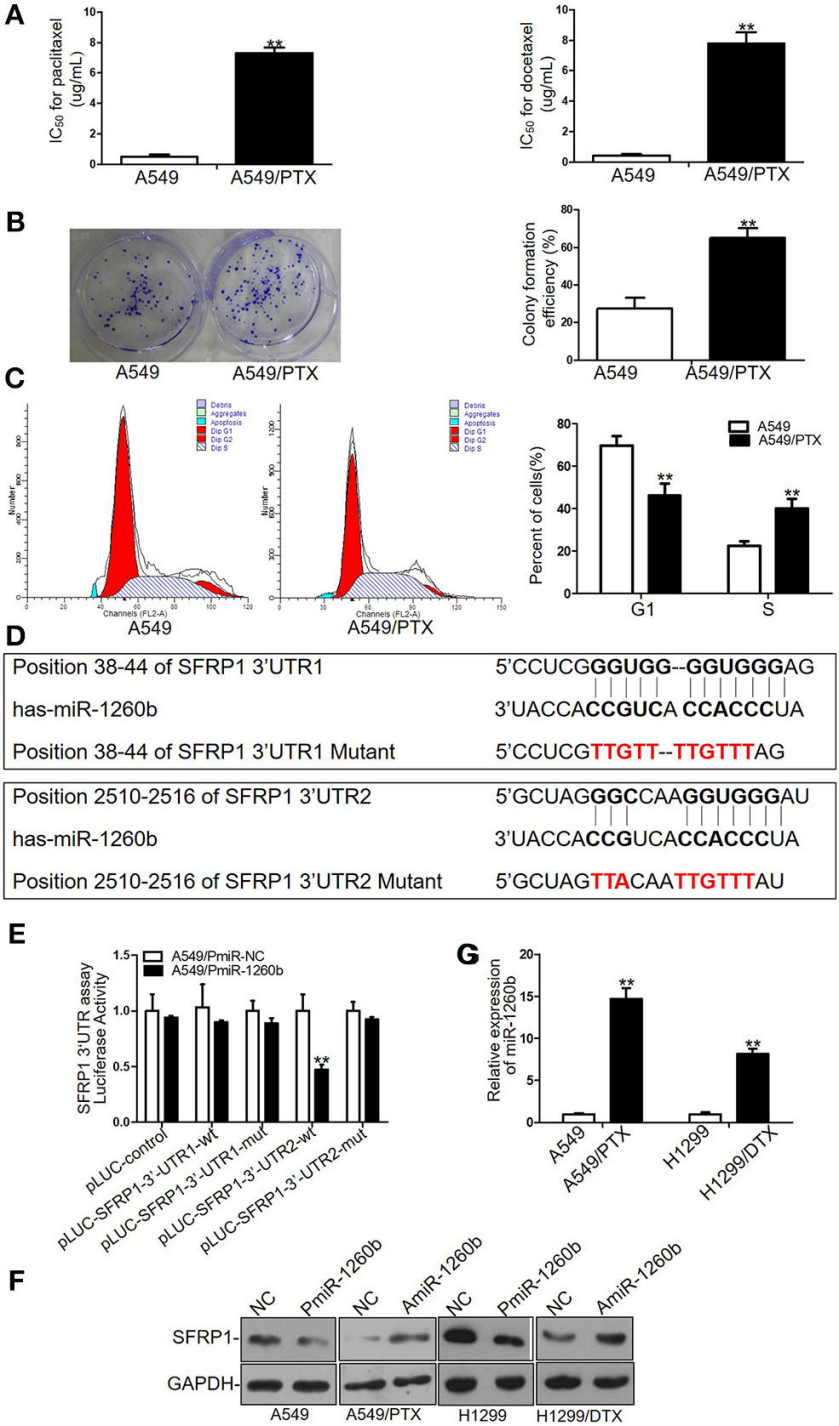
Different sensitivity to paclitaxel and docetaxel between A549/PTX cells and parental A549 cells and SFRP1 was a direct target of miR-1260b in LAD cells. **(A)** IC_50_ values for paclitaxel (left) and docetaxel (right) in A549 and A549/PTX cells as determined by MTT assays. **(B)** Proliferation ability of A549 and A549/PTX cells as determined by colony formation assays. **(C)** Cell cycle analysis of A549 and A549/PTX cells by flow cytometry. **(D)** Consensus sequences for miR-1260b in the SFRP1 3′-UTR were predicted by bioinformatics analysis. **(E)** A reporter vector containing the SFRP1 3′-UTR (wild-type or mutant constructs) was cotransfected with miR-1260b mimic in A549 cells, which were then analyzed using a dual-luciferase reporter assay. **(F)** SFRP1 protein levels in parental and taxane-resistant cells after transfection with miR-1260b mimic or inhibitor as determined by western blotting. **(G)** Expression of miR-1260b in A549/PTX and H1299/DTX cells compared with A549 and H1299 cells as indicated by qRT-PCR. miRNA abundance was normalized to the U6 RNA level. The experiment was repeated at least three times. Student *t*-test was employed to compare the differences between two groups. The data are represented as mean ± SD. **p < 0.01 compared with control group.

To investigate the biological mechanisms of chemoresistance in LAD cells, we previously established a paclitaxel-resistant cell line (A549/PTX) from parental A549 cells. Drug cytotoxicity in A549 and A549/PTX cells was evaluated by MTT assays. The IC_50_ values for paclitaxel were 0.71 ± 0.23 and 7.38 ± 0.89 μg/ml in A549 and A549/PTX cells, respectively (Figure 1A, left). The IC_50_ values of A549 and A549/PTX cells for docetaxel were 0.51 ± 0.31 and 8.34 ± 1.72 μg/ml, respectively, indicating that the A549/PTX cell line had acquired cross-resistance to docetaxel (Figure 1A, right). Colony formation assays revealed a significant enhancement of the proliferation ability of A549/PTX cells (Figure 1B). Flow-cytometric analyses revealed that compared with A549 cells, in A549/PTX cells, cells in the S phase were increased whereas those in the G1 phase were decreased (*p* < 0.01) (Figure 1C), while no significant differences were observed in apoptosis (data not shown).

Our previous study has shown that the expression level of SFRP1 was enormously down-regulated in A549/PTX cells compared with parental A549 cells (7). To elucidate the mechanism of SFRP1 downregulation, miRNA target prediction software (http://targetscan.org) was used to find potential miR-1260b target sequences in SFRP1 gene. The results indicated that SFRP1 contains two putative binding sites for miR-1260b at positions 38–44 and 2510–2516 in its 3′-UTR (Figure 1D). To evaluate whether miR-1260b effectively targets SFRP1, we performed luciferase reporter assays. The wild-type SFRP1 3′-UTR complementary to miR-1260b and respective mutant sequences were cloned into pLUC vectors, which were named pLUC-SFRP1-3′-UTR1 and pLUC-SFRP1-3′-UTR2 (corresponding to positions 38–44 and 2510–2516, respectively). A significant decrease in the relative luciferase activity was observed when the miR-1260b mimic was cotransfected with pLUC-SFRP1 3′-UTR2-wt, whereas the luciferase activity was unaffected in A549 cells transfected with the mutant 3′-UTRs and 3′-UTR1 reporters (Figure 1E). Next, we investigated the effect of miR-1260b on SFRP1 expression by western blot analyses. As shown in Figure 1F, miR-1260b downregulation increased SFRP1 expression in A549/PTX and H1299/DTX cells, whereas miR-1260b upregulation diminished SFRP1 expression in A549 and H1299 cells. To explore the mechanism of miR-1260b in regulating the chemoresistance of LAD cells, we employed a docetaxel-resistant H1299 cell line (H1299/DTX). QRT-PCR analysis showed that compared with A549 and H1299 cells, miR-1260b was upregulated 14.68-fold and 7.59-fold in A549/PTX and H1299/DTX cells, respectively (Figure 1G). These results demonstrated that miR-1260b directly targeted SFRP1. Furthermore, we hypothesized that miR-1260b may play an important role in taxane chemoresistance in LAD cells.

### Inhibition of miR-1260b Enhances *in vitro* Chemosensitivity of Taxane-Resistant LAD Cell Lines to Taxanes

**Figure 2:**
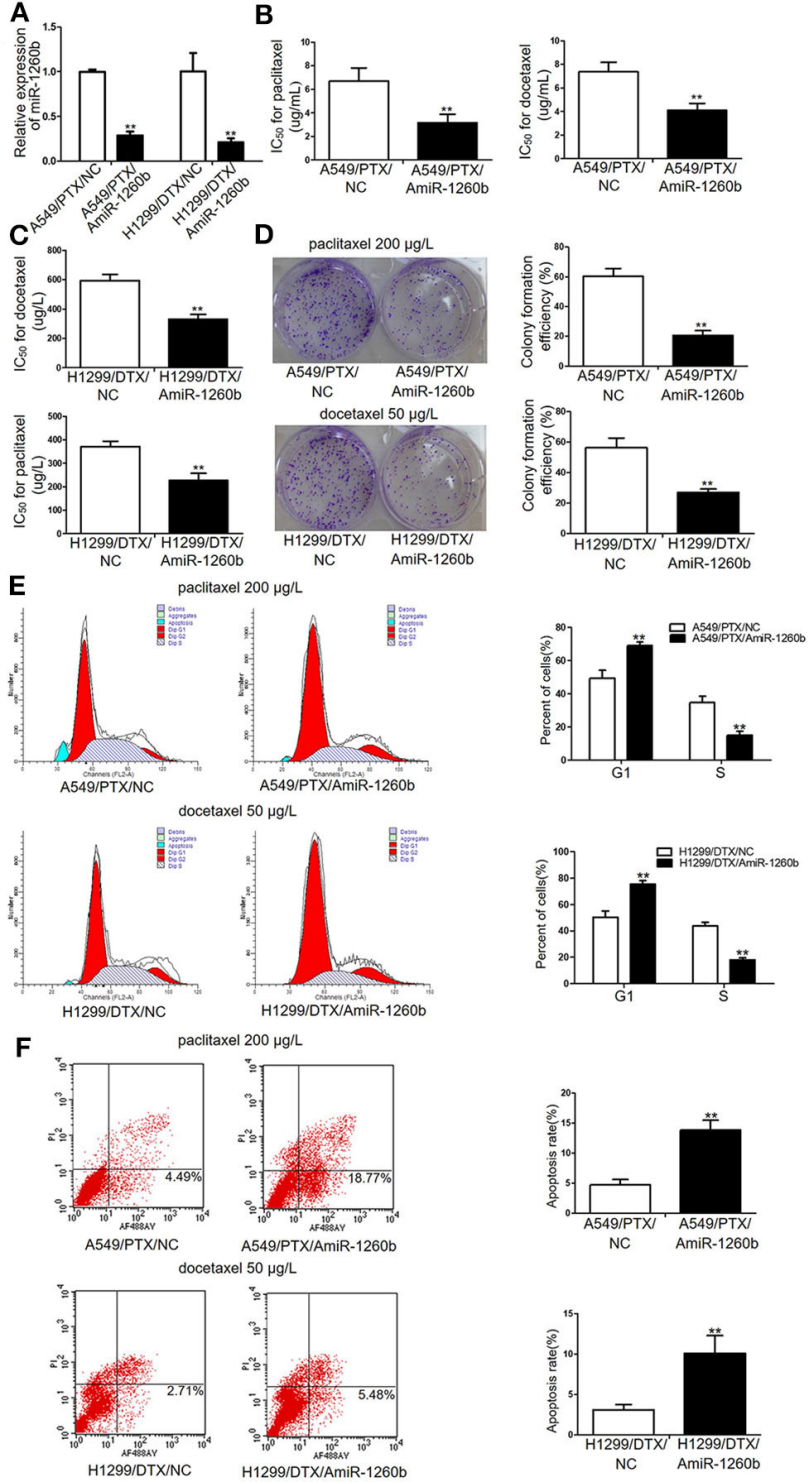
Inhibition of miR-1260b reversed the *in vitro* chemoresistance of A549/PTX and H1299/DTX cells to paclitaxel or docetaxel. **(A)** Expression levels of miR-1260b in A549/PTX and H1299/DTX cells transfected with mimic NC or miR-1260b inhibitor as determined by qRT-PCR. IC_50_ values for paclitaxel or docetaxel in A549/PTX **(B)** and H1299/DTX **(C)** cells transfected with mimic NC or miR-1260b inhibitor as determined by MTT assays. **(D)** Colony formation assays revealed proliferation ability of A549/PTX (upper) and H1299/DTX (lower) cells transfected with miR-1260b inhibitor, following treatment of paclitaxel (200 μg/L) or docetaxel (50 μg/L), respectively. Flow cytometric analysis indicated cell cycle **(E)** and apoptosis **(F)** in A549/PTX and H1299/DTX cells transfected with mimic NC or miR-1260b inhibitor, following treatment of paclitaxel (200 μg/L) or docetaxel (50 μg/L), respectively. Data between two groups were tested using independent sample *t*-test. The data are based on 3 to 5 independent experiments and are shown as mean ± SD. **p < 0.01 compared with NC group.

To investigate the relation between miR-1260b expression and the sensitivity of LAD cell lines to taxanes, A549/PTX and H299/DTX cells were transfected with miR-1260b inhibitor (AmiR-1260b) or AmiR-NC as a control. The transfection efficacy was confirmed by qRT-PCR (Figure 2A). Compared with A549/PTX/AmiR-NC cells, the IC_50_ values for paclitaxel and docetaxel in A549/PTX/AmiR-1260b cells were significantly decreased by 61.8 and 53.1%, respectively (*p* < 0.01, Figure 2B). Compared with H1299/DTX/AmiR-NC cells, the IC_50_ values for docetaxel and paclitaxel in H1299/DTX/AmiR-1260b cells were decreased by 39.2 and 33.1%, respectively (*p* < 0.01, Figure 2C). Colony formation assays revealed that the proliferation ability of A549/PTX and H299/DTX cells transfected with miR-1260b inhibitor was more significantly suppressed than that of NC-transfected cells when treated with paclitaxel (200 μg/L) or docetaxel (50 μg/L), respectively (*p* < 0.01, Figure 2D). The effects of miR-1260b on cell cycle and apoptosis were evaluated by flow cytometry. Following paclitaxel (200 μg/L) or docetaxel (50 μg/L) treatment, inhibition of miR-1260b in A549/PTX or H1299/DTX cells caused an increase in the percentage of cells in G1 phase and a decrease in the population in S phase, as well as a dramatic increase in the apoptosis rate (*p* < 0.01, Figures 2E,F). Thus, suppression of miR-1260b can reverse the chemoresistance of LAD cells for docetaxel and paclitaxel by suppressing their proliferation and promoting apoptosis.

### Ectopic miR-1260b Expression Reduces the *in vitro* Chemosensitivity of LAD Cells to Taxanes

**Figure 3:**
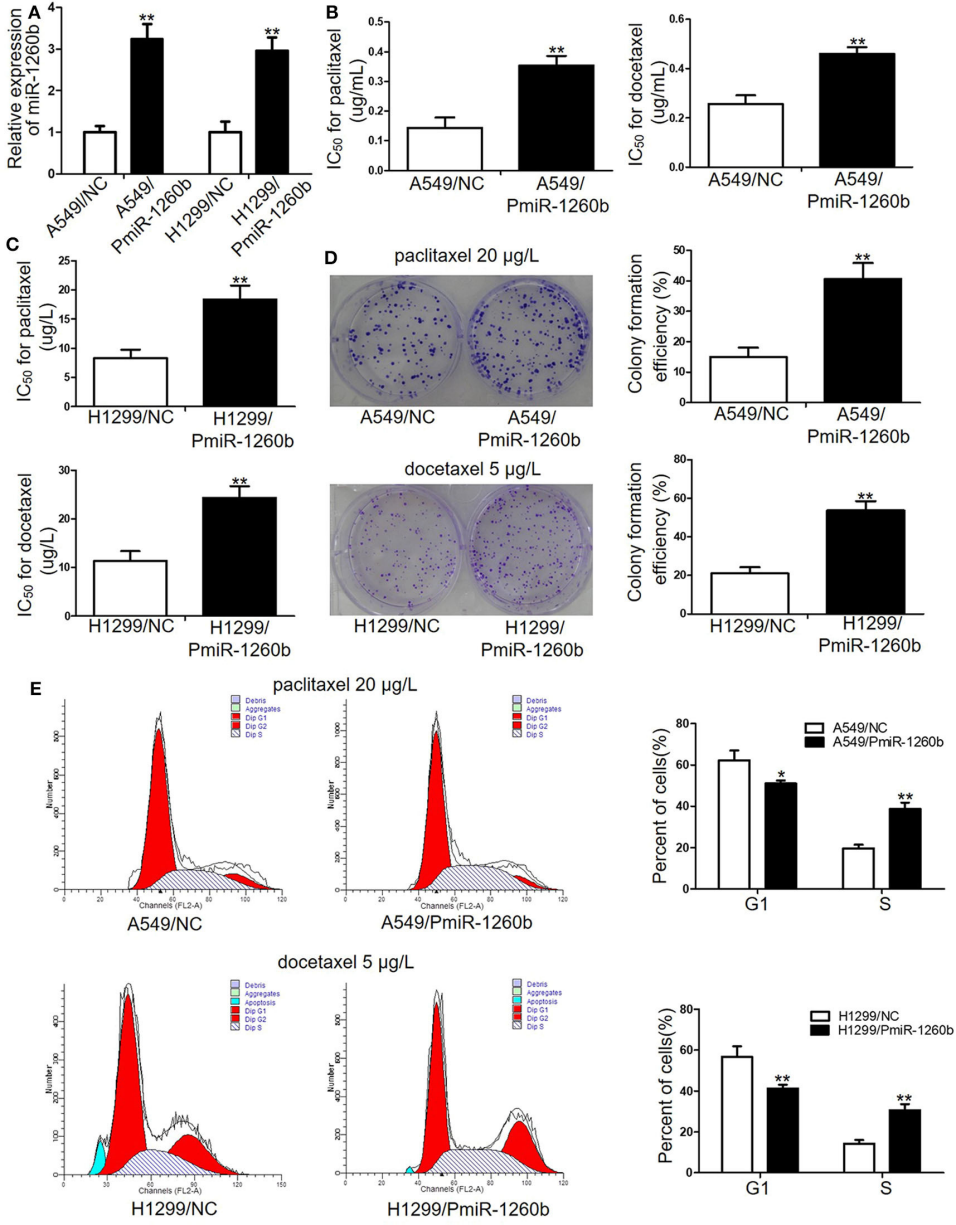
Ectopic miR-1260b expression decreased the chemosensitivity of A549 and H1299 cells to paclitaxel or docetaxel. **(A)** Expression levels of miR-1260b in A549 and H1299 cells transfected with mimic NC or miR-1260b mimic were determined by qRT-PCR assay. IC_50_ values for paclitaxel or docetaxel in A549 **(B)** and H1299 **(C)** cells transfected with mimic NC or miR-1260b mimic as determined by MTT assays. **(D)** Colony formation assays revealed proliferation ability of A549 (upper) and H1299 (lower) cells transfected with mimic NC or miR-1260b mimic following treatment of paclitaxel (20 μg/L) or docetaxel (5 μg/L), respectively. **(E)** Flow cytometry indicated cell cycle of A549 (upper) and H1299 (lower) cells transfected with mimic NC or miR-1260b mimic following treatment of paclitaxel (20 μg/L) or docetaxel (5 μg/L), respectively. Paired *t*-test was applied to compare the differences between two groups. Similar results were obtained in 3 independent experiments and are shown as mean ± SD. *p < 0.05; **p < 0.01 compared with NC group.

To determine whether upregulation of miR-1260b would affect the sensitivity of LAD cells to taxanes, A549 and H299 cells were transfected with miR-1260b mimic (PmiR-1260b). QRT-PCR confirmed the increased expression of miR-1260b in the LAD cells (Figure 3A). MTT assay results indicated that the IC_50_ values for paclitaxel and docetaxel were significantly increased by 54.8 and 47.6%, respectively, in A549/PmiR-1260b cells, and by 39.2 and 51.7%, respectively, in H1299/AmiR-1260b cells (*p* < 0.01, Figures 3B,C). However, compare IC_50_ for the drugs in miR-1260b inhibitor transfected cells and generated resistant cells a much lower resistance was reached. Next, we analyzed the effect of miR-1260b upregulation on the colony formation ability of LAD cells upon exposure to paclitaxel (20 μg/L) or docetaxel (5 μg/L). As shown in Figure 3D, upregulation of miR-1260b increased the proliferation ability of A549 and H1299 cells (*p* < 0.01). Moreover, compared with the NC group, enforced miR-1260b expression in A549 or H1299 cells resulted in an increased percentage of cells in the S phase, whereas the numbers of cells in the G1 phase were decreased (*p* < 0.01, Figure 3E). We did not observe changes in apoptosis. These results suggested that miR-1260b upregulation partially decreased chemosensitivity of LAD cells to taxanes.

### Effect of miR-1260b on Chemoresistance of Taxane-Resistant LAD Cells *in vivo*

**Figure 4:**
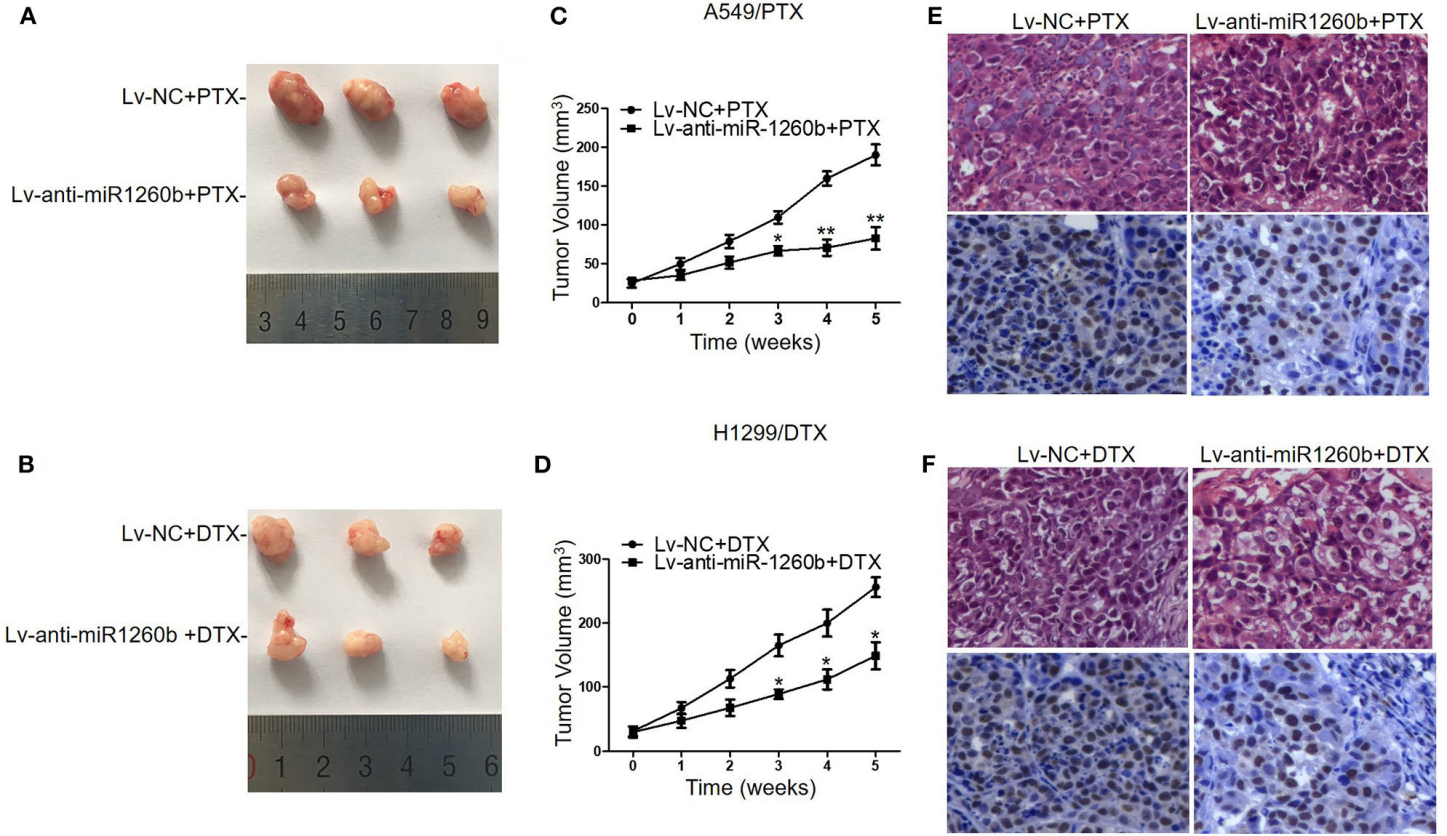
Inhibition of miR-1260b increased the chemosensitivity of A549/PTX and H1299/DTX cells to taxanes *in vivo*. Nude mice were subcutaneously injected with A549/PTX or H1299/DTX cells that had been infected with lentivirus carrying miR-1260b inhibitor and treated with paclitaxel (15 mg/kg) or docetaxel (1 mg/kg) when the mean tumor volume reached ~100 mm^3^. **(A)** Representative picture of tumors from A549/PTX/Lv-NC or A549/PTX/Lv-anti-miR1260b group with paclitaxel treatment. **(B)** Representative picture of tumors from H1299/DTX/Lv-NC or H1299/DTX/Lv-anti-miR1260b group with docetaxel treatment. **(C)** Growth curve of tumors derived from A549/PTX/Lv-NC or A549/PTX/Lv-anti-miR1260b cells. **(D)** Growth curve of tumors derived from H1299/DTX/Lv-NC or H1299/DTX/Lv-anti-miR1260b cells. Paired *t*-test was performed for comparisons between two groups. Each data point represents the mean ± SD of 7 mice. The three independent experiments gave similar results. *p < 0.05; **p < 0.01 compared with Lv-NC group. **(E)** H&E- (upper) and PCNA-stained (lower) sections of the transplanted tumors from A549/PTX/Lv-NC or A549/PTX/Lv-anti-miR1260b cells upon paclitaxel treatment. **(F)** H&E- (upper) and PCNA-stained (lower) sections of the transplanted tumors from H1299/DTX/Lv-NC or H1299/DTX/Lv-anti-miR1260b cells upon docetaxel treatment. Photomicrographs were taken at 400x magnification.

We next employed xenograft mouse models to explore the possible effect of miR-1260b on the chemosensitivity of LAD cells *in vivo*. A549/PTX and H1299/DTX cells were stably transfected with Lv-NC or Lv-anti-miR-1260b and subcutaneously injected into nude mice. QRT-PCR confirmed the decreased expression of miR-1260b in taxane-resistant LAD cells (Supplementary Figure 1). Approximately 10 days after inoculation, all mice developed tumors. When the tumor volume reached ~100 mm^3^, we treated mice bearing miR-1260b knocked down A549/PTX cell tumors, mice bearing miR-1260b knocked down H1299/DTX cell tumors, and mice bearing A549/PTX or H1299/DTX cell tumors (negative control, NC) with paclitaxel or docetaxel, respectively, as described in the materials and methods. As shown in Figures 4A–D, the tumor size and volume were decreased in the Lv-anti-miR-1260b group as compared to the NC group. Immunohistochemical analysis showed that the PCNA-positive rate was lower in the Lv-anti-miR-1260b group than in the NC group (Figures 4E,F). From these data, we concluded that miR-1260b inhibition can significantly increase the chemosensitivity of LAD cells to taxanes *in vivo*.

### miR-1260b Activates Wnt Pathway by Directly Targeting SFRP1 in LAD Cells

**Figure 5:**
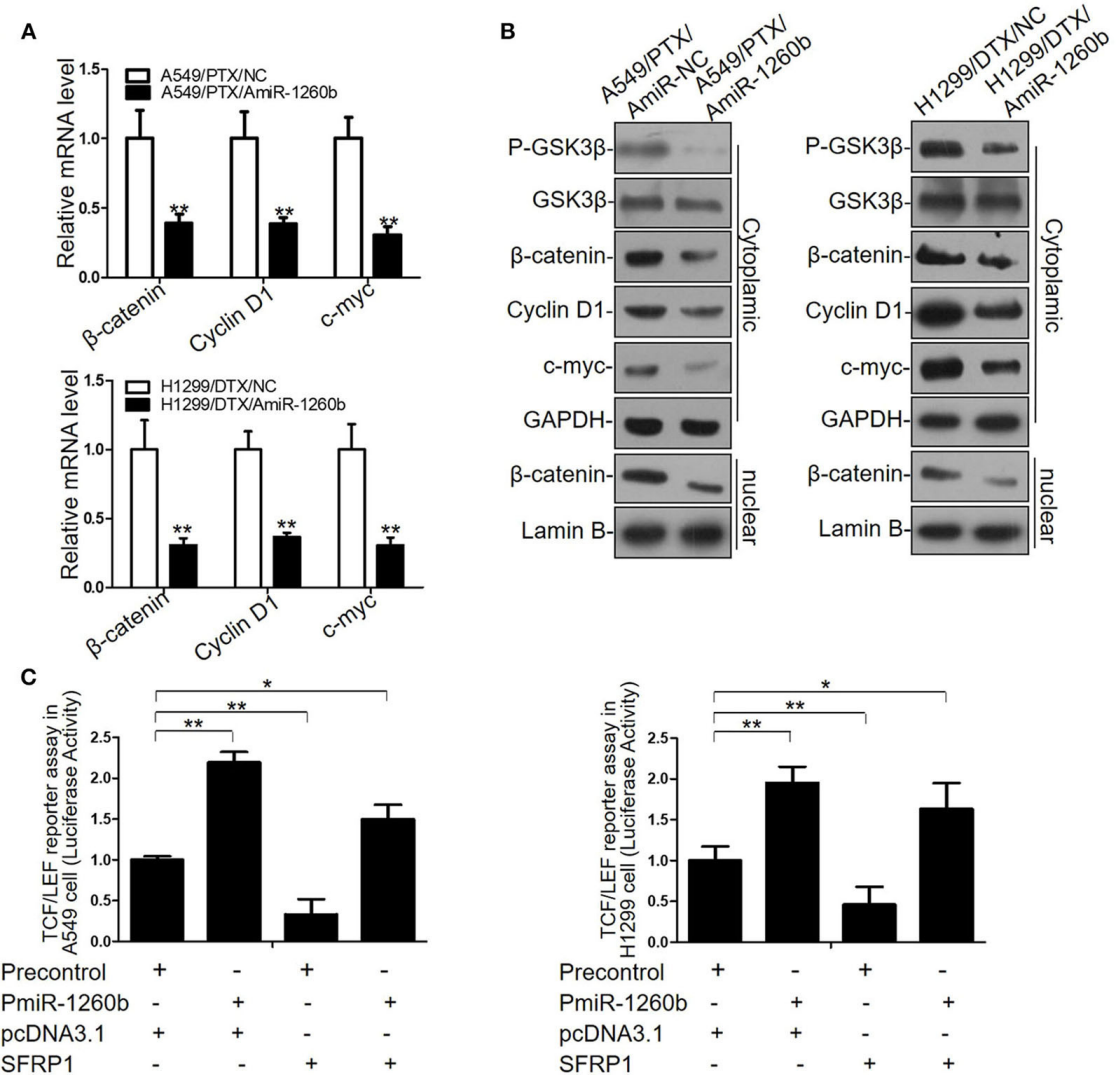
miR-1260b affected Wnt signaling activity by directly targeting SFRP1 in LAD Cells. **(A)** mRNA expression levels of β-catenin, cyclin D1, and c-myc in A549/PTX and H1299/DTX cells transfected with miR-NC or miR-1260b inhibitor as determined by qRT-PCR. **(B)** Protein expression levels of p-GSK3β, GSK3β, β-catenin, cyclin D1, and c-myc in A549/PTX and H1299/DTX cells transfected with miR-NC or miR-1260b inhibitor as determined by western blotting. **(C)** TCF/LEF reporter assay to assess the effect of miR-1260b on Wnt signaling activity in A549 (left) and H1299 (right) cells. Data are represented as mean ± SD of three independent experiments (Student *t*-test). *p < 0.05; **p < 0.01.

We previously reported that SFRP1 modulates taxane resistance in LAD cell lines by inactivating the Wnt signaling pathway (7). Thus, we explored the effect of miR-1260b on Wnt signaling activity in the A549/PTX and H1299/DTX cell lines. Based on our previous data and the results shown in Supplementary Figure 2, we determined that the Wnt signaling pathway was activated in A549/PTX and H1299/DTX cells. Compared with the parental cell lines, GSK3β phosphorylation was significantly increased in both A549/PTX and H1299/DTX cells. In addition, β-catenin accumulation in the cytoplasm and nuclei was confirmed by qRT-PCR and western blot assays. Furthermore, transcript levels of target genes of β-catenin, including c-Myc and cyclin D1, were increased in the two taxane-resistant LAD cell lines. We next evaluated whether downregulation of miR-1260b could inactivate the Wnt signaling pathway. miR-1260b inhibition suppressed the expression levels of p-GSK3β, β-catenin, cyclin D1, and c-Myc in both A549/PTX and H1299/DTX cells (Figures 5A,B). A TCF/LEF reporter assay of Wnt signaling pathway activity revealed that miR-1260b significantly increased the TCF reporter activity in A549 and H1299 cells, whereas the presence of pcDNA-SFRP1 only partially attenuated this effect by miR-1260b (*p* < 0.01, Figure 5C). The above results suggest that miR-1260b may not only suppress SFRP1 expression by directly binding its 3′-UTR, thereby affecting Wnt signaling activity, but also upregulate Wnt signaling activity via other signaling pathways in the LAD cells.

### Effects of miR-1260b on Epithelial-Mesenchymal Transition (EMT) in LAD Cells *in vitro* and *in vivo*

**Figure 6:**
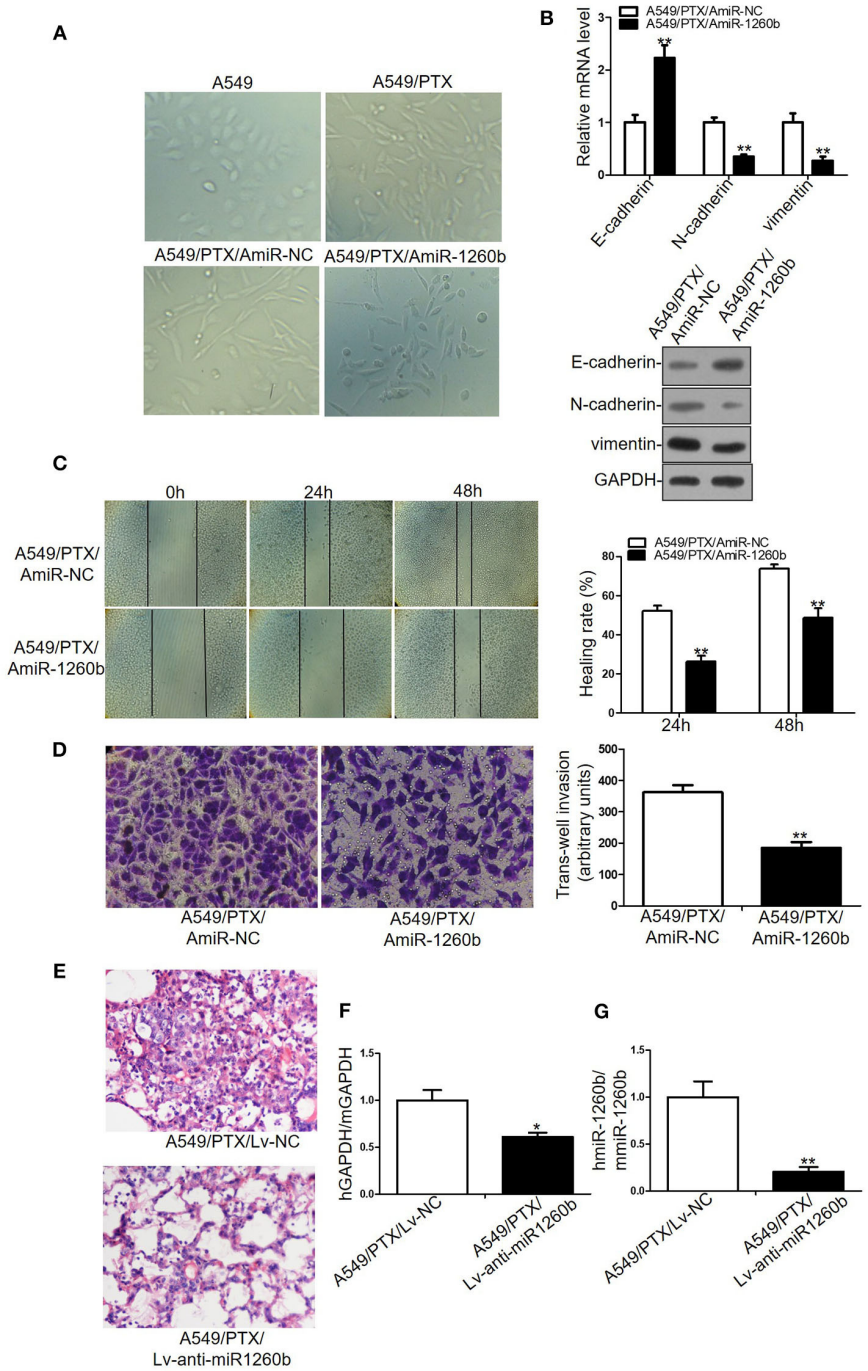
Inhibition of miR-1260b reversed the EMT phenotype and suppressed the invasive ability of A549/PTX cell line. **(A)** The phenotype of A549, A549/PTX, A549/PTX/AmiR-NC, A549/PTX/AmiR-1260b cells. Photomicrographs were taken at 200x magnification. **(B)** mRNA and protein expression levels of E-cadherin, N-cadherin, and vimentin in A549/PTX/AmiR-NC and A549/PTX/AmiR-1260b cells as determined by qRT-PCR (left) and western blotting (right). Cell migration and invasion capacities of A549/PTX/AmiR-NC and A549/PTX/AmiR-1260b cells as determined by wound healing **(C)** and invasion **(D)** assays, respectively. Photomicrographs were taken at 200x magnification. **(E)** Eight weeks after tail vein injection of A549/PTX/Lv-NC or A549/PTX/Lv-anti-miR1260b cells, metastatic potential was determined by H&E staining. **(F)** Human-specific GAPDH (hGAPDH) expression relative to mouse-specific GAPDH (mGAPDH) in circulating tumor cells as determined by qRT-PCR. **(G)** Human-miR-1260b (hmiR-1260b) expression relative to mouse-miR-1260b (mmiR-1260b) in circulating tumor cells as determined by qRT-PCR. The data are represented as mean ± SD of triplicate samples performed in two independent samples (Student *t*-test). *p < 0.05; **p < 0.01 compared with control group.

EMT plays a role in tumor recurrence, metastasis, and drug resistance. Wnt pathway activation has been shown to be associated with EMT in numerous tumor models. Previous studies have revealed that A549/PTX and H1299/DTX cell lines showed EMT phenotype, including morphologic changes, EMT marker expression, and increased migratory and invasive capacities (13, 16). In the present study, we explored the relationship between miR-1260b expression and EMT features. Morphological study showed that A549/PTX cells presented marked morphologic changes including loss of cell polarity, increased formation of pseudopodia and leading to elongated, irregular fibroblastoid appearance. A549/PTX cells transfected with miR-1260b inhibitor displayed a more epithelial appearance compared with control group (Figure 6A). As shown in Figure 6B and Supplementary Figure 3A, the expression of the epithelial marker E-cadherin was increased, whereas that of the mesenchymal markers including vimentin and N-cadherin was decreased in A549/PTX and H1299/DTX cells transfected with the miR-1260b inhibitor. Transwell invasion and wound healing assays showed that miR-1260b downregulation significantly inhibited the invasive and migratory capacities of A549/PTX and H1299/DTX cells when compared with parental cells (Figures 6C,D and Supplementary Figures 3B,C). We next examined the role of miR-1260b in the metastatic potential of A549/PTX cells *in vivo*. H&E staining revealed that compared with the A549/PTX/Lv-anti-miR1260b group, lung tissues in the A549/PTX/Lv-NC group had more multinucleate, huge cells and scattered lymphocytes (Figure 6E). Concentration of circulating tumor cells were significantly decreased in A549/PTX/Lv-anti-miR1260 cell-injected nude mice (Figures 6F,G, *p* < 0.05). Together, these data demonstrated that miR-1260b inhibition can reverse the EMT phenotype of taxane-resistant cells.

### High Expression of miR-1260b in LAD Tissues Is Associated With Decreased SFRP1 Expression, Taxane-Resistance, and Poor Prognosis

**Figure 7:**
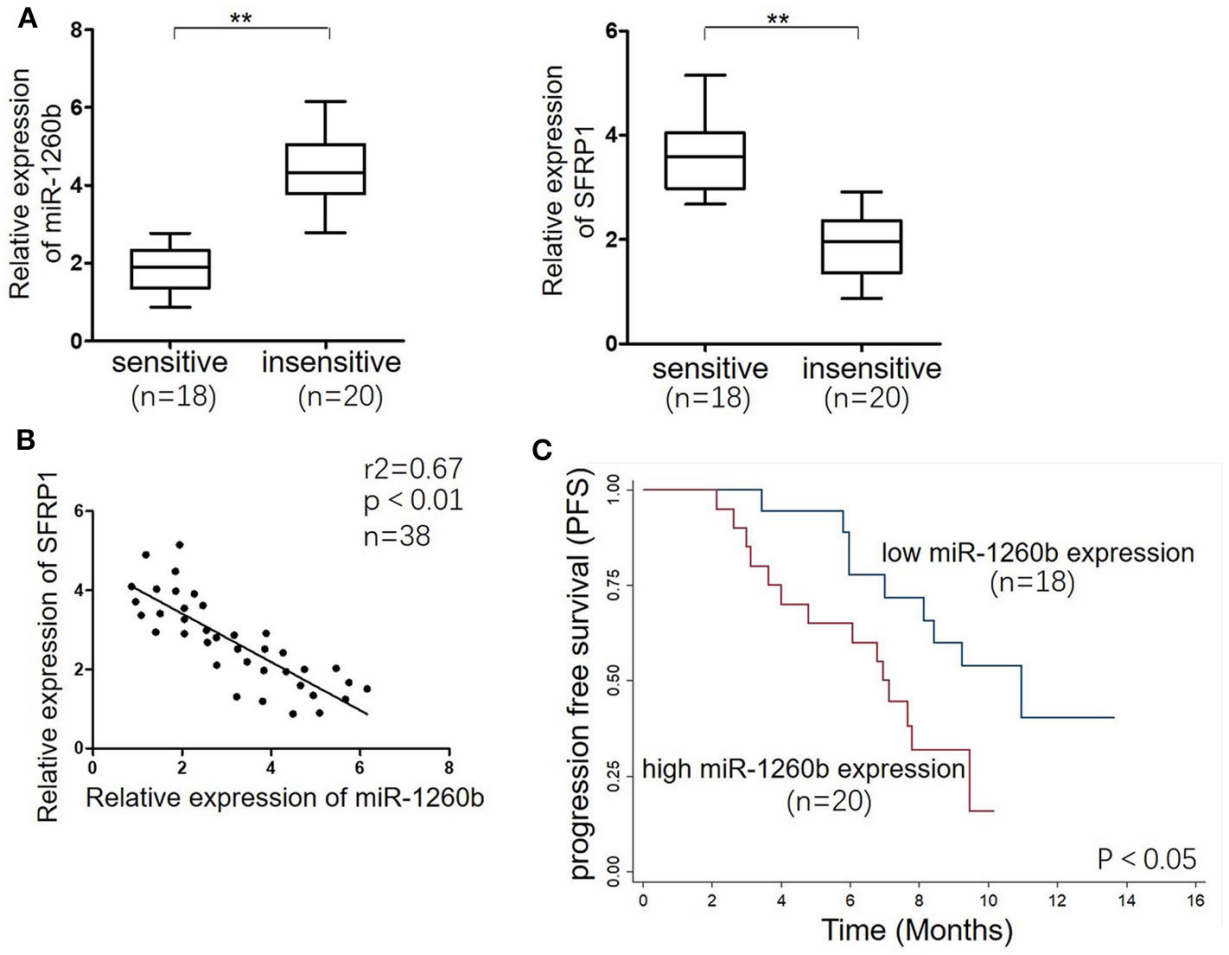
miR-1260b was associated with SFRP1 expression and poor prognosis in LAD tumor tissue samples. **(A)** mRNA expression levels of miR-1260b (left) and SFRP1 (right) in sensitive (*n* = 18) and insensitive (*n* = 20) LAD tissues as detected by qRT-PCR. U6 RNA and GAPDH were used as internal controls. The data express as the mean ± SD (Student *t*-test). **p < 0.01. **(B)** Linear regression analysis revealed an inverse correlation between miR-1260b and SFRP1. **(C)** Kaplan–Meier survival analysis of LAD patients according to the level of miR-1260b expression in tumor tissues. The *p*-value was determined with the log-rank test.

To investigate the relationship between miR-1260b and SFRP1 expression *in vivo* further, tumor tissue samples were obtained from 38 patients with advanced LAD. Based on the patient response to taxane-based chemotherapies, tumors were classified as “sensitive” (CR + PR) or “insensitive” (SD + PD). There was no difference in age, sex, and smoking statues between patients with sensitive tumors and patients with insensitive tumors. Half of the patients in each group were smokers. Figure 7A shows that the relative levels of miR-1260b were significantly higher in “insensitive” tumors (*n* = 20) than in the “sensitive” tumors (*n* = 18), whereas SFRP1 mRNA expression showed opposite trends. The inverse correlation between miR-1260b and SFRP1 mRNA expression was confirmed by linear regression analysis (Figure 7B). Further, Kaplan–Meier analysis revealed the association between miR-1260b expression and patient survival; LAD patients with high miR-1260b expression had a poorer progression-free survival than those with low miR-1260b expression (*p* < 0.05, Figure 7C). These data suggested that the miR-1260b level is inversely correlated with SFRP1 expression in LAD patients and is linked to taxane-based chemotherapy resistance.

## Discussion

The present study provided new insights into the molecular mechanisms underlying chemoresistance in LAD. By targeting SFRP1, miR-1260b can modulate the chemosensitivity and EMT phenotype of taxane-resistant LAD cells via affecting Wnt signaling pathway activity. In addition, miR-1260b is closely associated with SFRP1 expression in advanced LAD tumors and the patient response to taxane-based chemotherapy.

Tumor cells acquire resistance to taxanes via various mechanisms, including aberrant DNA methylation of key genes, abnormal activation of certain pathways, ectopic expression of oncogenic microRNAs, and dysregulation of major proteins involved in drug transport and metabolism (17). As a novel member of the SFRP family, SFRP1 has been confirmed to exert inhibitory effects on tumor cell growth, invasion, and chemoresistance (18). Our previous study indicated that SFRP1 is significantly downregulated in taxane-resistant LAD cells and tissues (7). miRNAs participate in tumor development by targeting multiple cancer-related genes (19). In this study, we identified SFRP1 as a direct target of miR-1260b based on miRNA target prediction database search and luciferase reporter assays. In addition, the SFRP1 level was inversely correlated with miR-1260b expression. In line with this observation, Hirata et al. documented that SFRP1 may be transcriptionally silenced by miRNA-1260b in renal cancer cells (20). Intriguingly, SFRP1 is also targeted by other miRNAs, including miR-27a, miR-1180, and miR-454-3p (21–23). miRNAs can simultaneously modulate multiple genes, via imperfect base-pairing and different binding sites, thereby forming a complex regulatory network (24). In addition, evidence suggests that SFRP1 transcription is frequently regulated by epigenetic events, such as DNA methylation alterations and chromatin histone modifications, in various tumor models (25). These mechanisms remain to be validated in future research.

miRNAs participate in the regulation of tumor genes, cellular processes, tumor aggressiveness, and chemoresistance (26). A recent study indicated that miR-1260b acts as an important oncogenic miRNA in prostate cancer development (27). Increased miR-1260b expression has been also observed in lung cancer tissues, particularly in patients with positive lymph nodes (28). Xia Y. et al. has also proved that miR-1260b promoted xenograft tumor formation in NSCLC (29). In addition, miR-1260b overexpression promoted the proliferation and invasion of hepatocellular carcinoma cells (30). However, the function of miR-1260b on chemotherapeutic sensitivity in LAD remained unclear. In the current study, both *in-vitro* and *in*-*vivo* experiments indicated that downregulation of miR-1260b could resensitize taxane-resistant LAD cells to chemotherapy by suppressing cell proliferation and promoting apoptosis and G1 phase arrest, whereas ectopic miR-1260b expression in LAD cells had the opposite effects. Therefore, we suggest that miR-1260b may function as an oncogene to affect the sensitivity of LAD cells to taxanes. To the best of our knowledge, this is the first report documenting the effect of miR-1260b on chemoresistance in LAD cells.

SFRP1 contains a cysteine-rich domain homologous to frizzleds, the seven-pass trans-membrane cell surface receptors for Wnt signaling pathway (18). SFRP1 acts as a soluble inhibitor of Wnt signaling by inhibiting the binding of Wnt ligands to frizzled, causing β-catenin phosphorylation and reducing β-catenin levels. An increasing body of evidence indicates that aberrant activation of Wnt signaling may contribute to abnormal cell proliferation, invasion, and chemoresistance (31). Activated Wnt/β-catenin signals lead to translocation of β-catenin into the nucleus, thereby regulating the expression of downstream targets, including cyclinD1 and c-Myc (32). CyclinD1 has been implicated in chemoresistance by virtue of its influence on G1/S restriction, and c-Myc is a key regulator of apoptosis (33). Consistent with these functions, we previously demonstrated that SFRP1 overexpression could inhibit Wnt signaling by decreasing the expression of β-catenin target genes, as mentioned above (7). In the current study, miR-1260b silencing had similar effects as ectopic SFRP1 expression in TCF/LEF reporter and western blot assays. Therefore, we suggest that both miR-1260b silencing and SFRP1 expression restoration can reverse taxane chemoresistance of LAD cell lines by inactivating the Wnt pathway.

Several potential chemoresistance mechanisms involving EMT have been suggested (34, 35). It is well-known that EMT occurs during tumor migration and invasion, and facilitates tumor progression (36). Furthermore, mesenchymal-type cancer cells are significantly less sensitive to chemotherapeutic agents than epithelial-type cancer cells are, suggesting an influence of the cell phenotype on the sensitivity to therapeutic agents (13, 37). In line with these reports, we found EMT features in taxane-resistant cell lines, such as loss of epithelial marker and gain of mesenchymal marker expression. Amounting evidence indicates that miRNA alterations and abnormal Wnt/β-catenin signaling activation account not only for chemoresistance, but also EMT characteristics of lung cancer cells. Yang et al. reported that miR-1246 might promote lung tumor cell metastasis by regulating the expression of GSK-3β and β-catenin (38). Zheng et al. discovered that miR-495 represses EMT progression in non-small-cell lung cancer cells, potentially by inactivating the Wnt/β-catenin pathway (39). Our previous study revealed that SFRP1 restoration could reverse the EMT process by inhibiting the Wnt pathway in A549 cells (40). The current study revealed that miR-1260b knockdown resulted in the reversal of EMT characteristics, including morphological changes, marker protein expression and migratory potential, in A549/PTX and H1299/DTX cells. Further, SFRP1 expression was elevated in response to miR-1260b downregulation in LAD tissues, and we found an association between miR-1260b expression and progression-free survival in LAD patients, suggesting that miR-1260b and SFRP1 might function as important regulators in the chemosensitivity and EMT phenotype of LAD cells.

**Figure 8:**
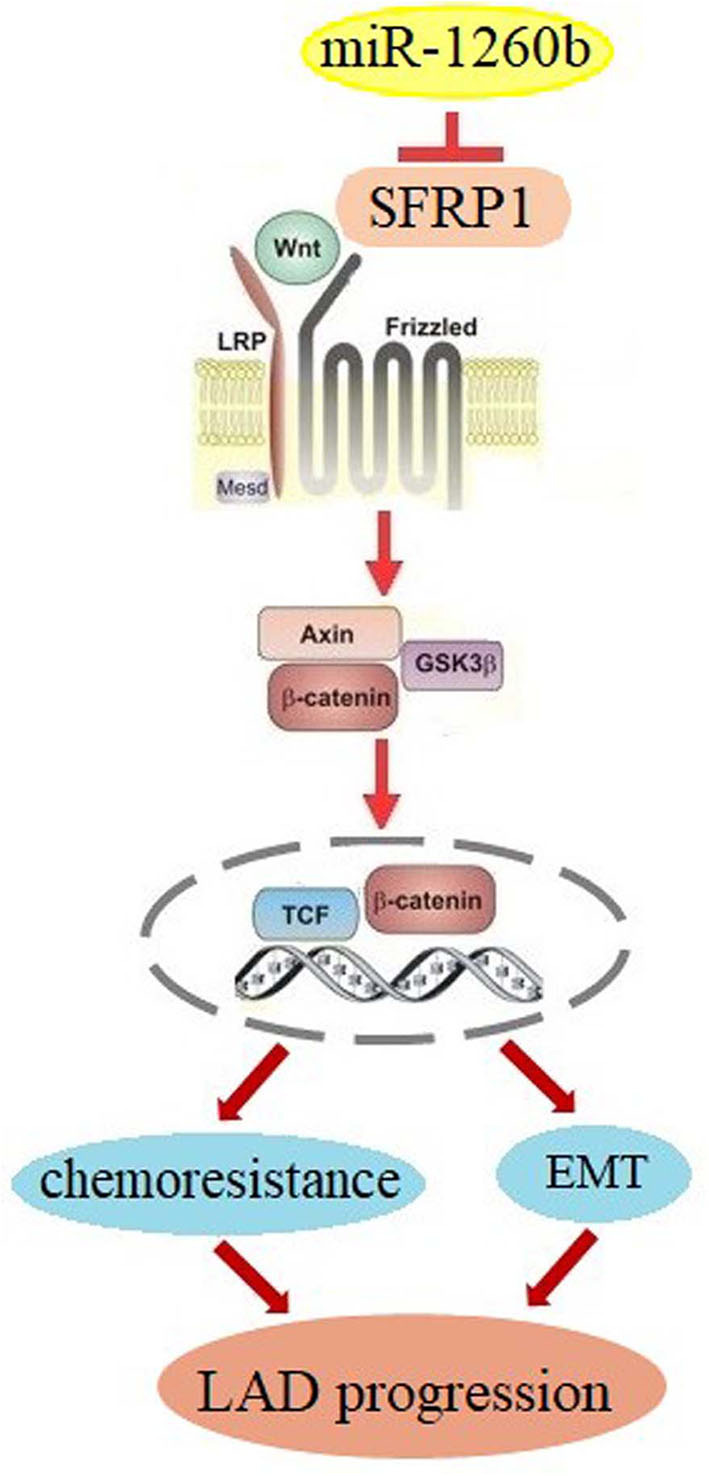
Schematic diagram for miR-1260b modulation of chemoresistance in NSCLC. miR-1260b regulates chemoresistance by targeting SFRP1 and thereby activating Wnt signaling.

In summary, our study demonstrated for the first time that miR-1260b, a new onco-miRNA, can modulate resistance to taxanes and the EMT phenotype in human LAD cells via SFRP1 inhibition and Wnt signaling activation (Figure 8). Thus, targeting the miR-1260b/SFRP1/Wnt signaling axis may be a promising strategy for the treatment of chemoresistant LAD in the future. However, our findings remain to be confirmed in studies using more LAD cell lines and clinical tissue samples.

## Data Availability Statement

The raw data supporting the conclusions of this article will be made available by the authors, without undue reservation.

## Ethics Statement

The studies involving human participants were reviewed and approved by the Ethics Committee of Affiliated Hospital of Jiangsu University. The patients/participants provided their written informed consent to participate in this study. The animal study was reviewed and approved by the Institutional Animal Care and Use Committee of Jiangsu University.

## Author Contributions

JR, DW, HH, XL, XZ, and JL designed the study. JR, DW, HH, and XL collated the data, carried out data analyses, and wrote the initial draft of the manuscript. JL contributed to the critical reading and revision of the manuscript. All authors reviewed and approved the final manuscript.

## Conflict of Interest

The authors declare that the research was conducted in the absence of any commercial or financial relationships that could be construed as a potential conflict of interest.

## Supplementary Material

Supplementary Figure 1 QRT-PCR assay determined expression levels of miR-1260b in A549/PTX and H1299/DTX cells infected with lentivirus carrying miR-1260b inhibitor.

Supplementary Figure 2 The Wnt signaling pathway was activated in H1299/DTX cells. **(A)** mRNA expression levels of β-catenin, cyclin D1, and c-Myc in H1299 and H1299/DTX cells as determined by qRT-PCR. **(B)** Protein expression levels of p-GSK3β, GSK3β, β-catenin, cyclin D1, and c-Myc in H1299 and H1299/DTX cells as determined by western blotting. Results represented the average of three independent experiments (mean ± SD). ^**^*p* < 0.01 compared with H1299 cells.

Supplementary Figure 3 Inhibition of miR-1260b modulated the EMT phenotype and decreased the migratory ability of H1299/DTX cells. **(A)** mRNA and protein expression levels of E-cadherin, N-cadherin, and vimentin in H1299/DTX/AmiR-NC and H1299/DTX/AmiR-1260b cells as determined by qRT-PCR (left) and western blotting (right). Cell migration and invasion capacities of H1299/DTX/AmiR-NC and H1299/DTX/AmiR-1260b cells as determined by wound healing **(B)** and invasion **(C)** assays, respectively. Photomicrographs were taken at 200x magnification. Data are presented as mean ± SD based on at least three independent experiments (Student *t*-test). ^*^*p* < 0.05; ^**^*p* < 0.01 compared with NC group.

Supplementary Table 1 Primer sequences used for Real-time PCR.
