# Supplementary figures and images for: miR-1260b Activates Wnt Signaling by Targeting Secreted Frizzled-Related Protein 1 to Regulate Taxane Resistance in Lung Adenocarcinoma
Source: Front Oncol. 2020 Nov 5;10:557327. doi: 10.3389/fonc.2020.557327 (PMC7674592; doi:10.3389/fonc.2020.557327)

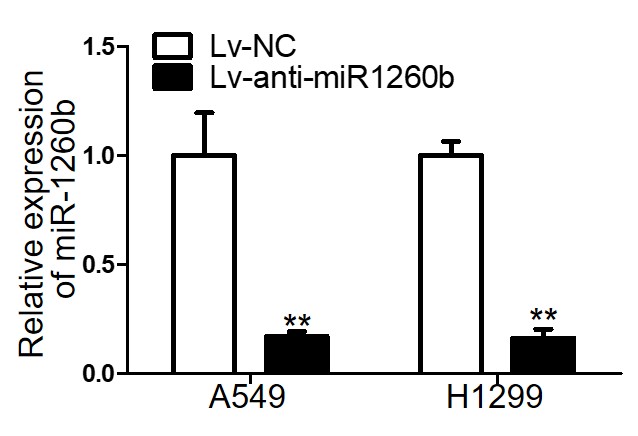

Supplement: Supplementary Figure 1 — QRT-PCR assay determined expression levels of miR-1260b in A549/PTX and H1299/DTX cells infected with lentivirus carrying miR-1260b inhibitor. [file Image_1.JPEG]

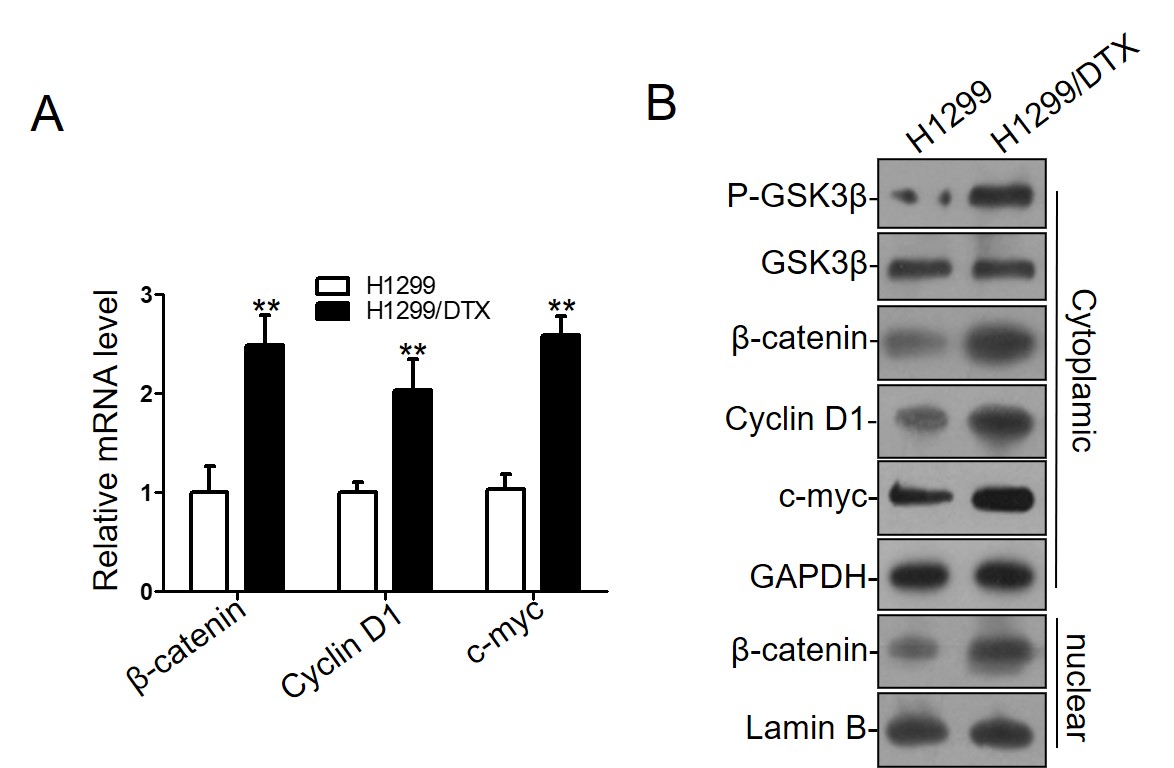

Supplement: Supplementary Figure 2 — The Wnt signaling pathway was activated in H1299/DTX cells. (A) mRNA expression levels of β-catenin, cyclin D1, and c-Myc in H1299 and H1299/DTX cells as determined by qRT-PCR. (B) Protein expression levels of p-GSK3β, GSK3β, β-catenin, cyclin D1, and c-Myc in H1299 and H1299/DTX cells as determined by western blotting. Results represented the average of three independent experiments (mean ± SD). **p < 0.01 compared with H1299 cells. [file Image_2.JPEG]

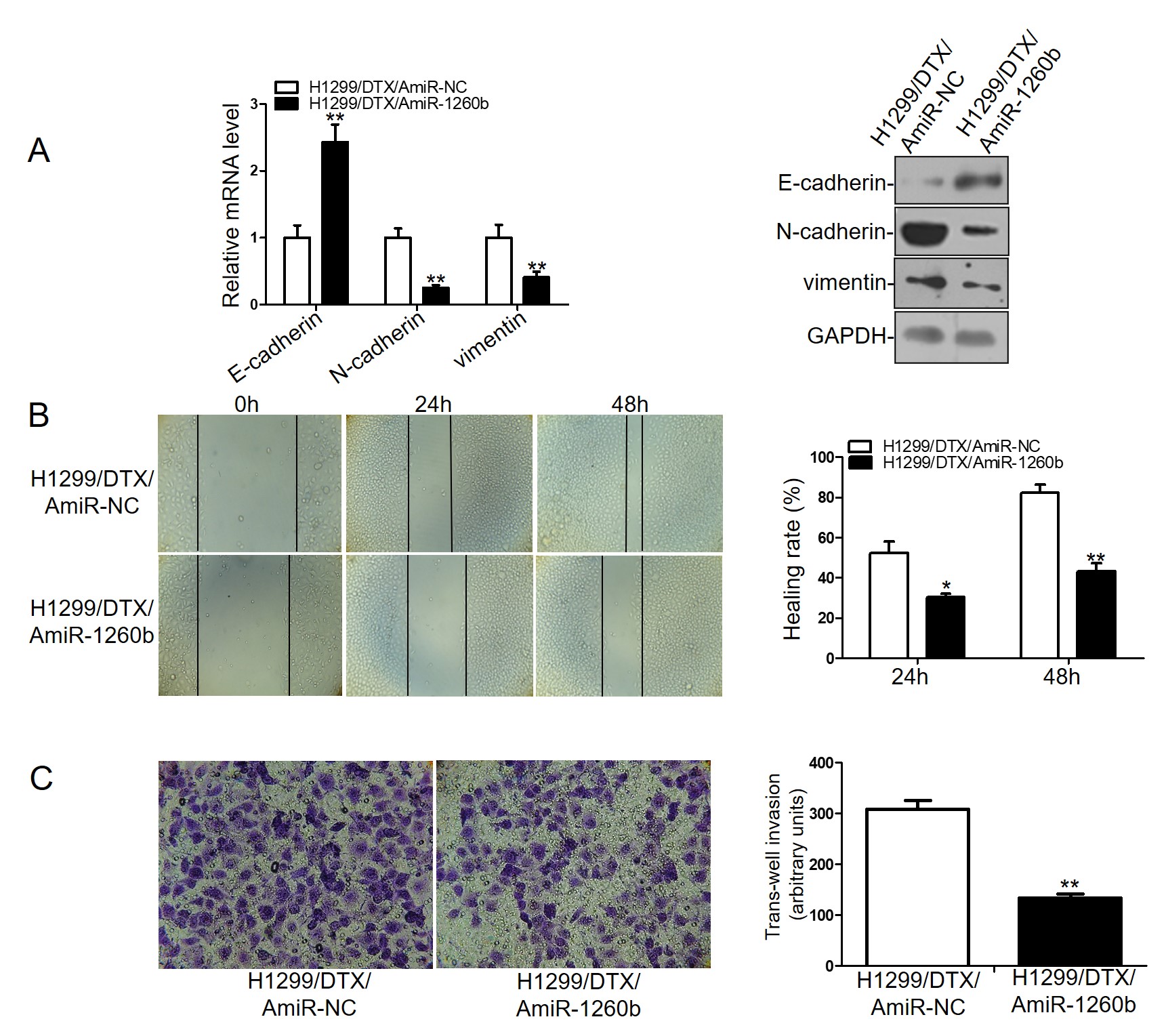

Supplement: Supplementary Figure 3 — Inhibition of miR-1260b modulated the EMT phenotype and decreased the migratory ability of H1299/DTX cells. (A) mRNA and protein expression levels of E-cadherin, N-cadherin, and vimentin in H1299/DTX/AmiR-NC and H1299/DTX/AmiR-1260b cells as determined by qRT-PCR (left) and western blotting (right). Cell migration and invasion capacities of H1299/DTX/AmiR-NC and H1299/DTX/AmiR-1260b cells as determined by wound healing (B) and invasion (C) assays, respectively. Photomicrographs were taken at 200x magnification. Data are presented as mean ± SD based on at least three independent experiments (Student t-test). *p < 0.05; **p < 0.01 compared with NC group. [file Image_3.JPEG]
