# Supplementary material for: miR-1260b Activates Wnt Signaling by Targeting Secreted Frizzled-Related Protein 1 to Regulate Taxane Resistance in Lung Adenocarcinoma
Source: Front Oncol. 2020 Nov 5;10:557327. doi: 10.3389/fonc.2020.557327 (PMC7674592; doi:10.3389/fonc.2020.557327)
Supplement: Supplementary Table 1 — Primer sequences used for Real-time PCR. [file Table_1.docx]

**SUPPLEMENTARY TABLE 1.** Primer sequences used for Real-time PCR.

| mRNA | Primers |
| --- | --- |
| SFRP1-  3’UTR1  SFRP1-  3’UTR2 | Forward: 5’-AAACTAGCGGCCGCTAGTCCTCGGGTGGGGTGGGAGT-3’  Reverse: 5’-CTAGACTCCCACCCCACCCGAGGACTAGCGGCCGCTAGTTT-3’  Forward: 5’-AAACTAGCGGCCGCTAGTGCTAGGGCCAAGGTGGGATT-3’  Reverse: 5’-CTAGAATCCCACCTTGGCCCTAGCACTAGCGGCCGCTAGTTT-3’ |
| β-catenin | Forward: 5’- AAGACATCACTGAGCCTGCCAT -3’  Reverse: 5’- CGATTTGCGGGACAAAGGGCAA -3’ |
| c-Myc | Forward: 5’- ACCACCAGCAGCGACTCTGA -3’  Reverse: 5’- TCCAGCAGAAGGTGATCCAGACT -3’ |
| cyclin-D1 | Forward: 5’- AATGACCCCGCACGATT -3’  Reverse: 5’- GCACAGAGGGCAACGAAGG -3’ |
| E-cadherin | Forward: 5’- CATTTCCCAACTCCTCTCCTGGC -3’  Reverse: 5’- ATGGGCCTTTTTCATTTTCTGGG -3’ |
| Vimentin | Forward: 5’- AGTTCAAGAACACCCGCACCAAC -3’  Reverse: 5’- CAGGAAGCGCACCTTGTCGATGT -3’ |
| N-cadherin | Forward: 5’- CTTCAGGCGTCTGTAGAGGCTTC -3’  Reverse: 5’- TGCACATCCTTCGATAAGACTGC -3’ |
| GAPDH | Forward: 5’- GCACCGTCAAGGCTGAGAAC -3’  Reverse: 5’- TGGTGAAGACGCCAGTGGA -3’ |
